# Supplementary material for: Effects of Extraction Methods on Phenolic Content in the Young Bamboo Culm Extracts of Bambusa beecheyana Munro
Source: Molecules. 2022 Apr 6;27(7):2359. doi: 10.3390/molecules27072359 (PMC9000241; doi:10.3390/molecules27072359)

## HPLC Chromatograms of standards

Figure S1. Chromatogram of p-coumaric acid

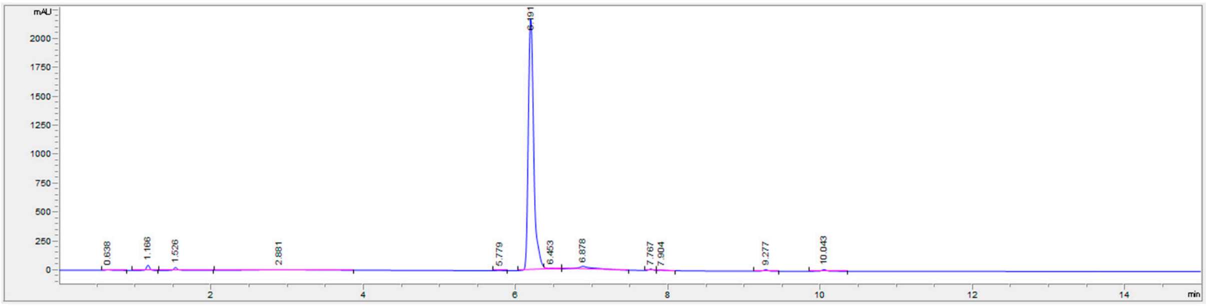


Figure S2. Chromatogram of 4-methoxycinnamic acid

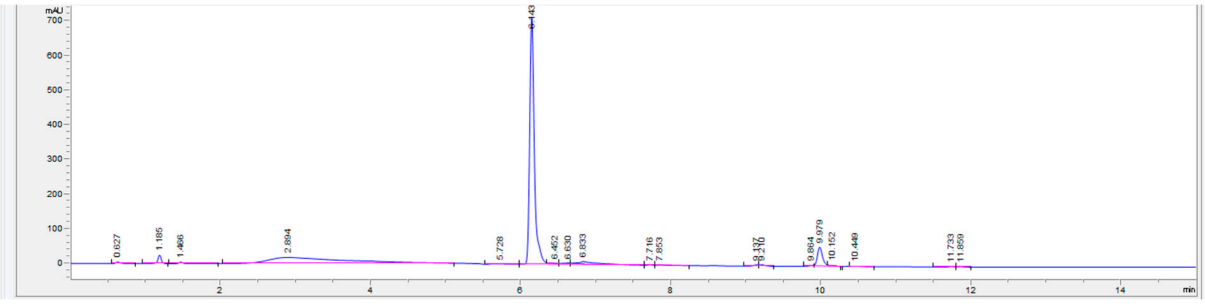


## HPLC chromatograms of all the extracts

Figure S3. Chromatogram of BBMR

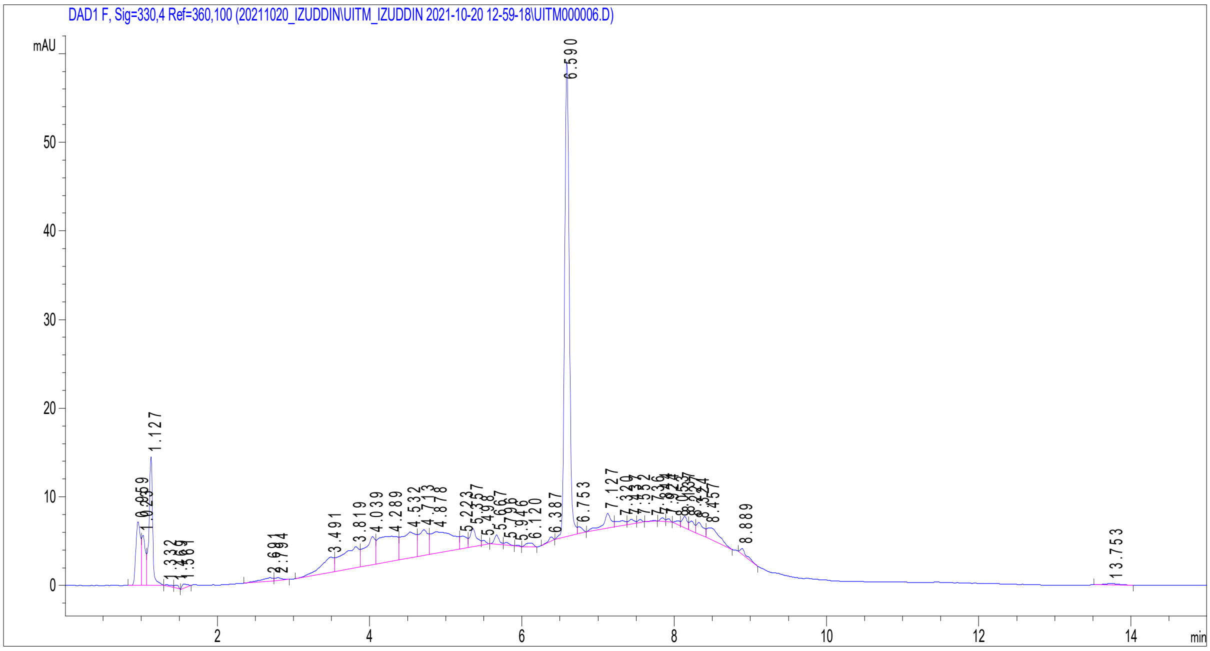

Figure S4. Chromatogram of BBHR

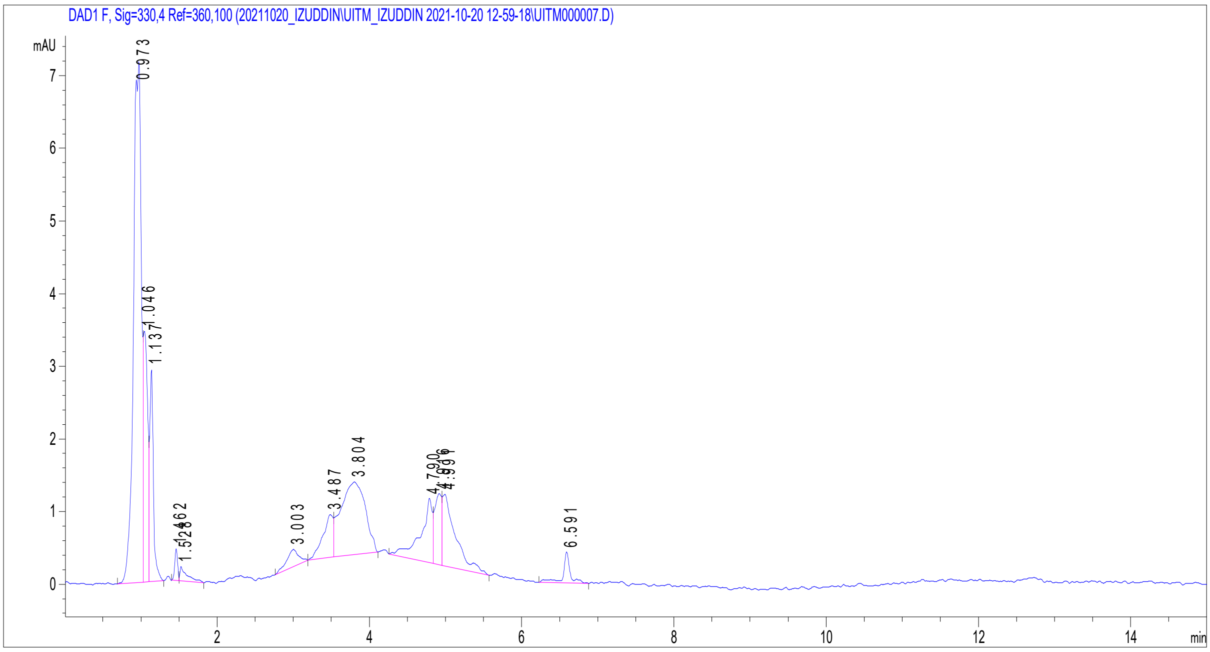


Figure S5. Chromatogram of BBES

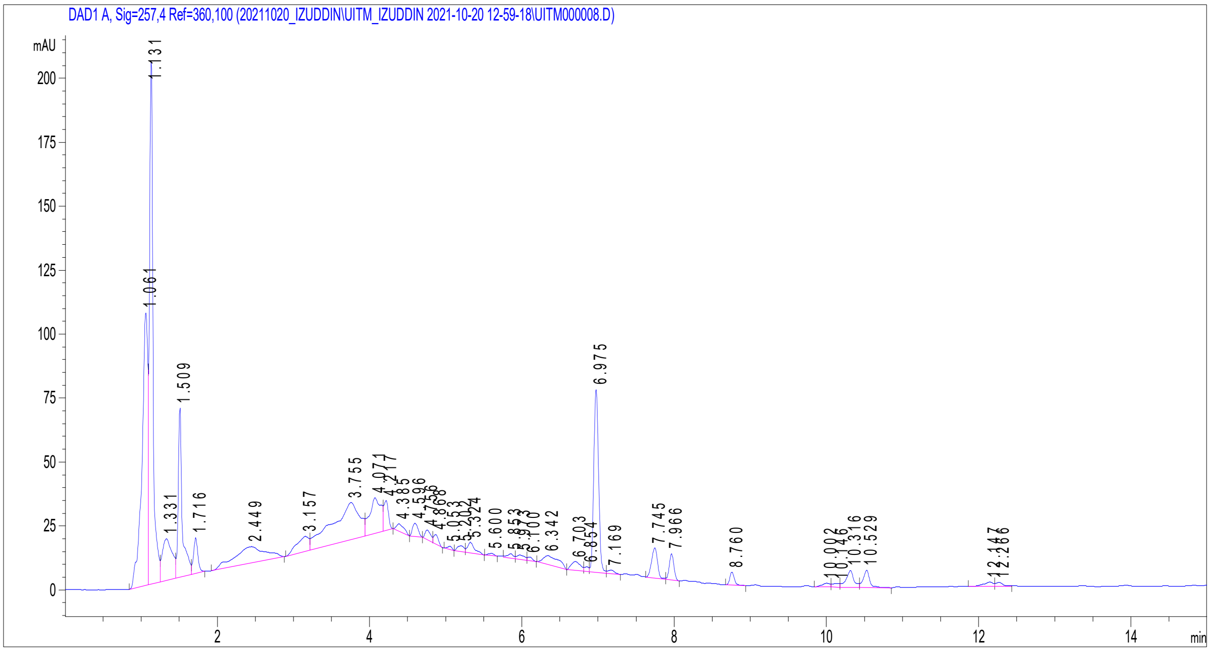

Figure S6. Chromatogram of BBMS

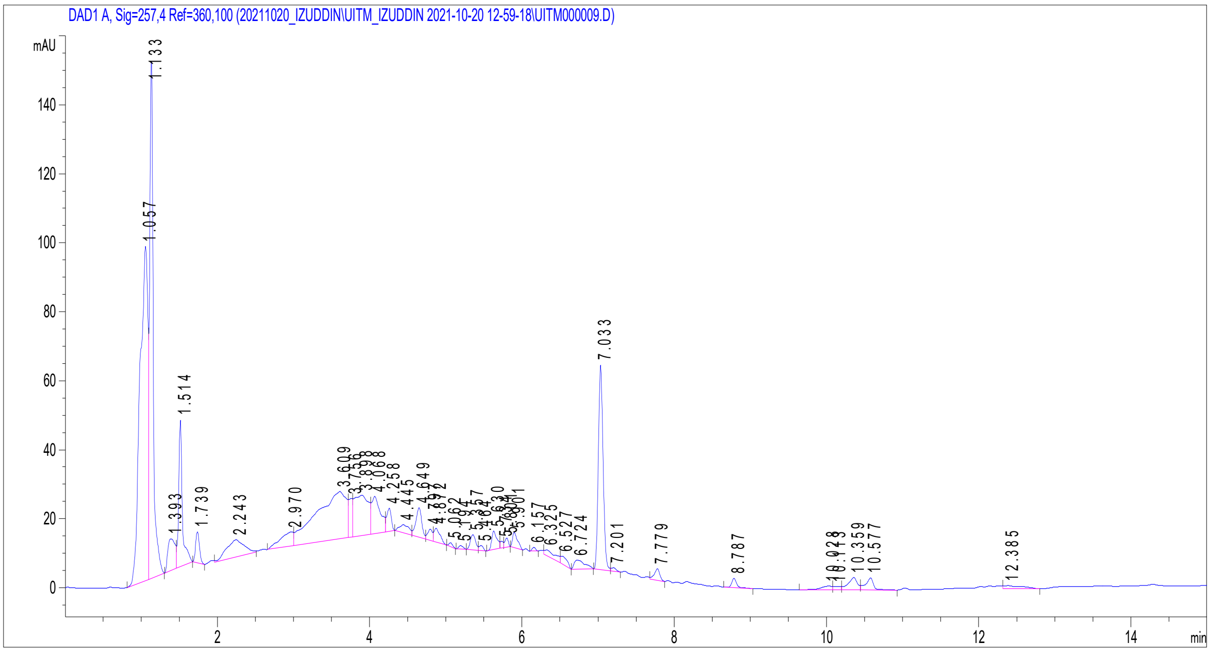


Figure S7. Chromatogram of BBHS

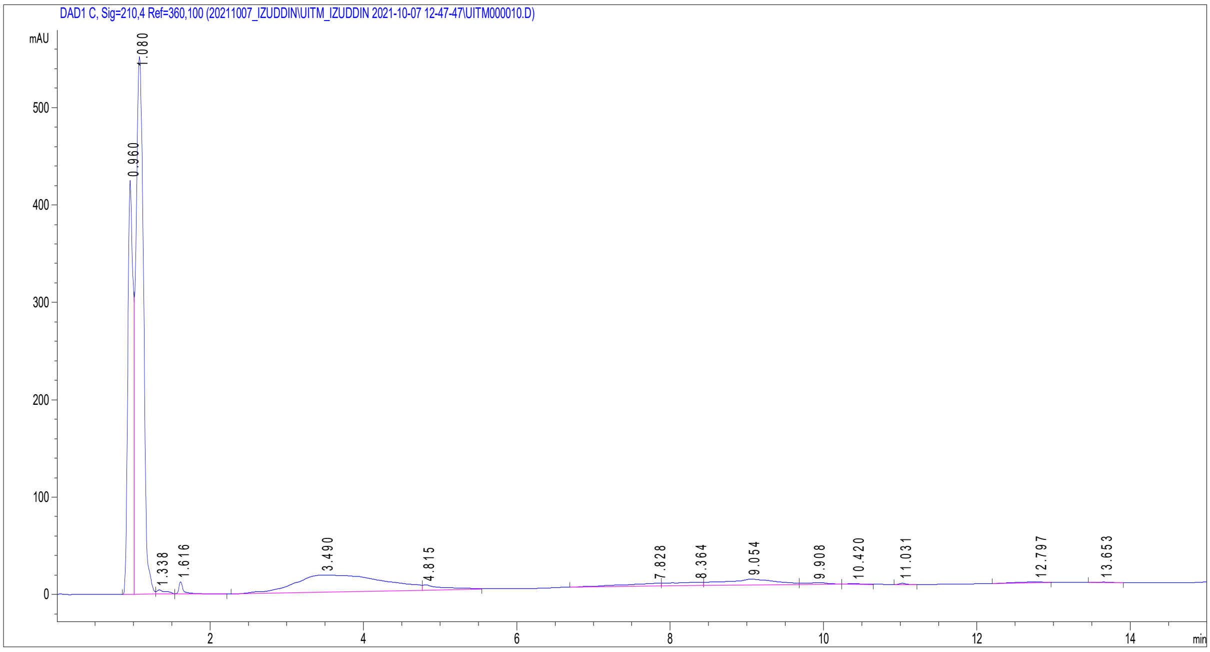

Figure S8. Chromatogram of BBEU20

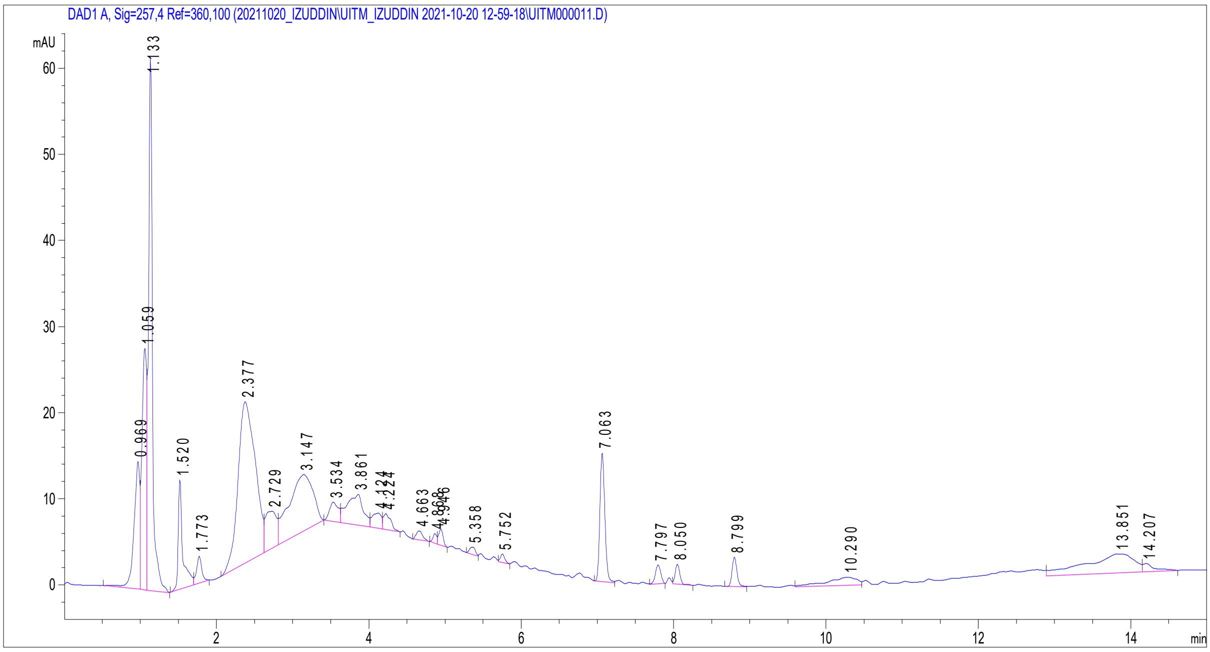


Figure S9. Chromatogram of BBEU40

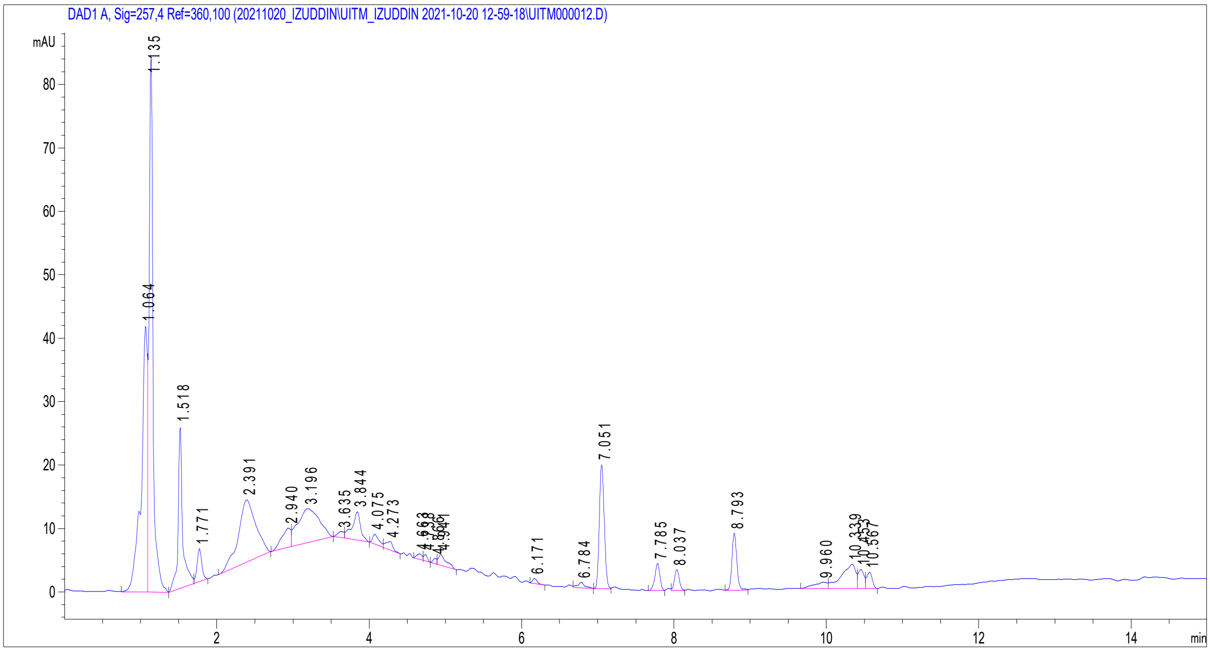

Figure S10. Chromatogram of BBEU60

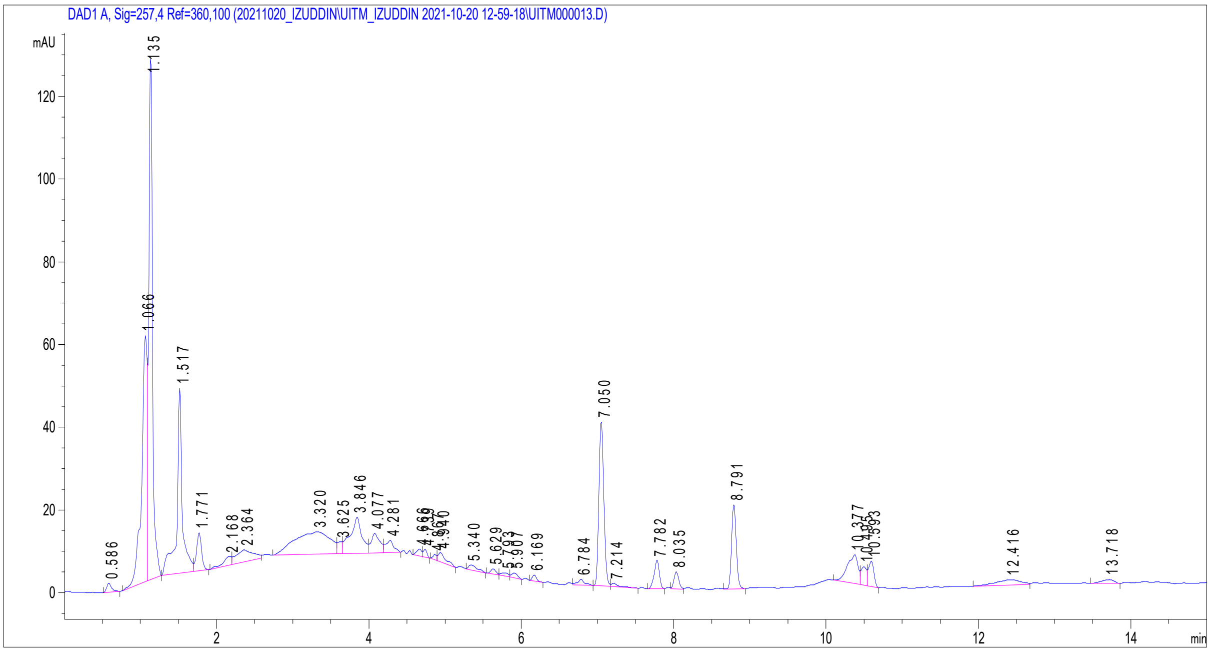


Figure S11. Chromatogram of BBMU20

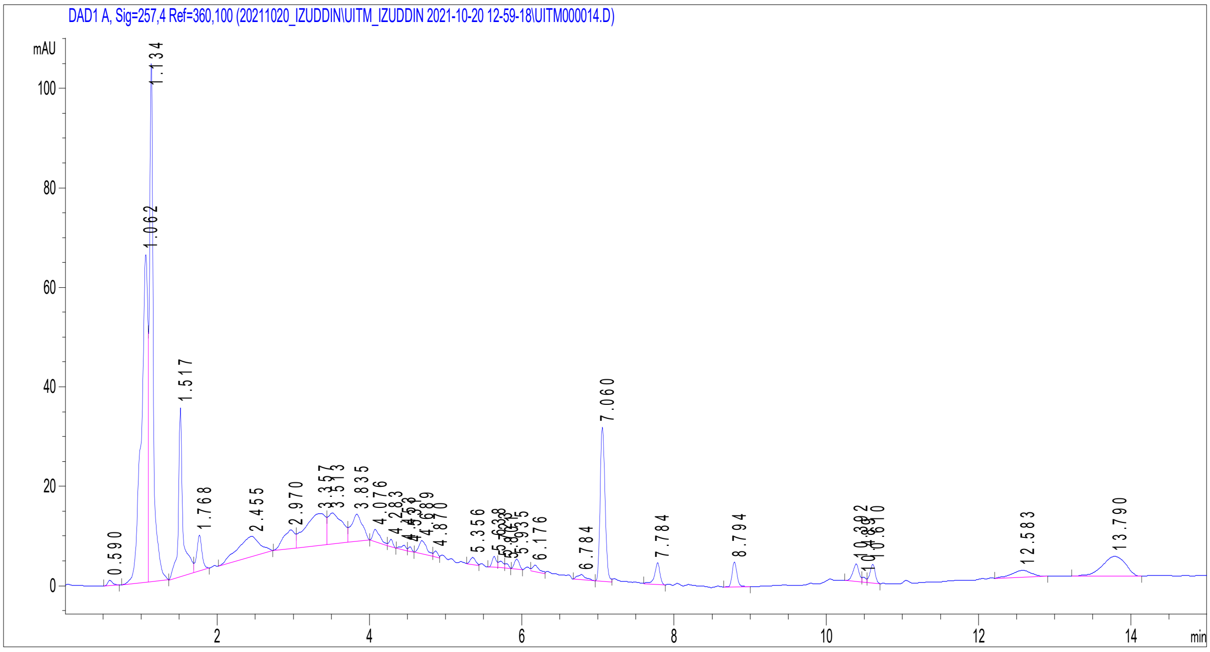

Figure S12. Chromatogram of BBMU40

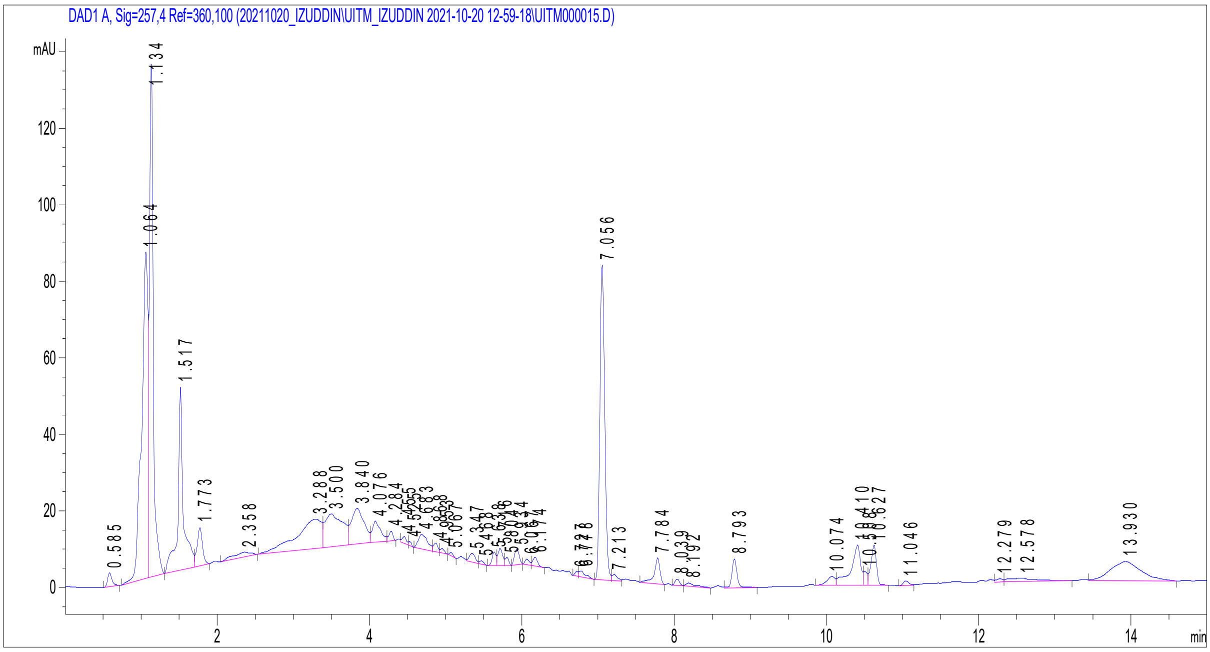


Figure S13. Chromatogram of BBMU60

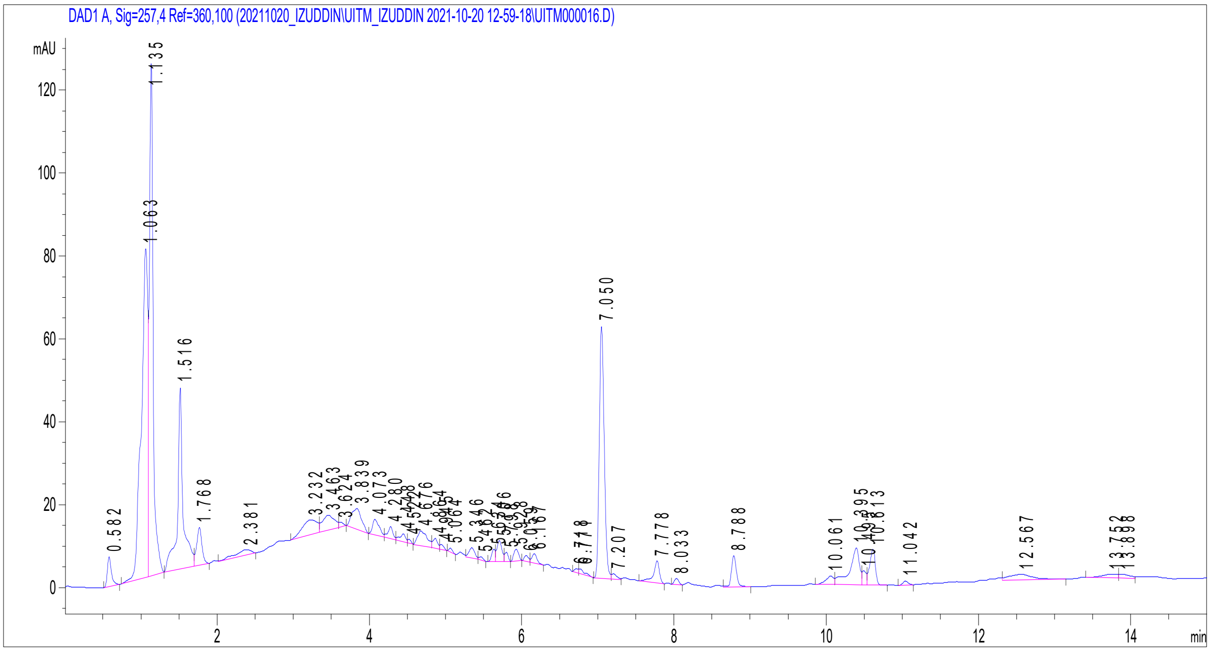

Figure S14. Chromatogram of BBHU20

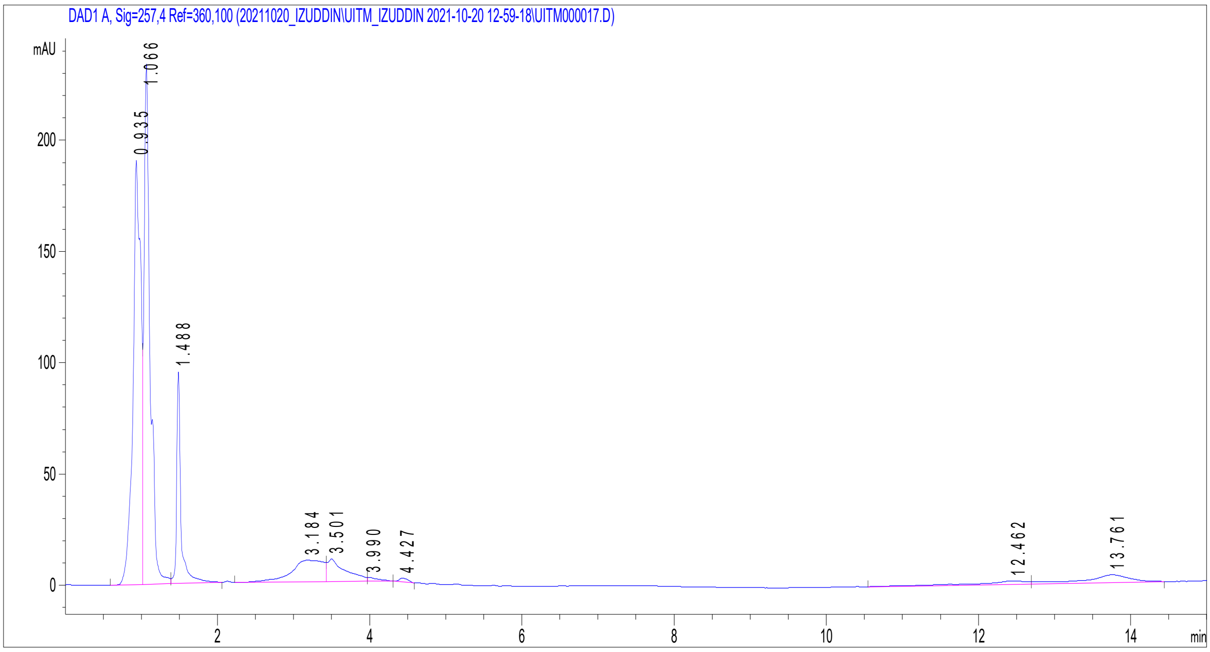


Figure S15. Chromatogram of BBHU40

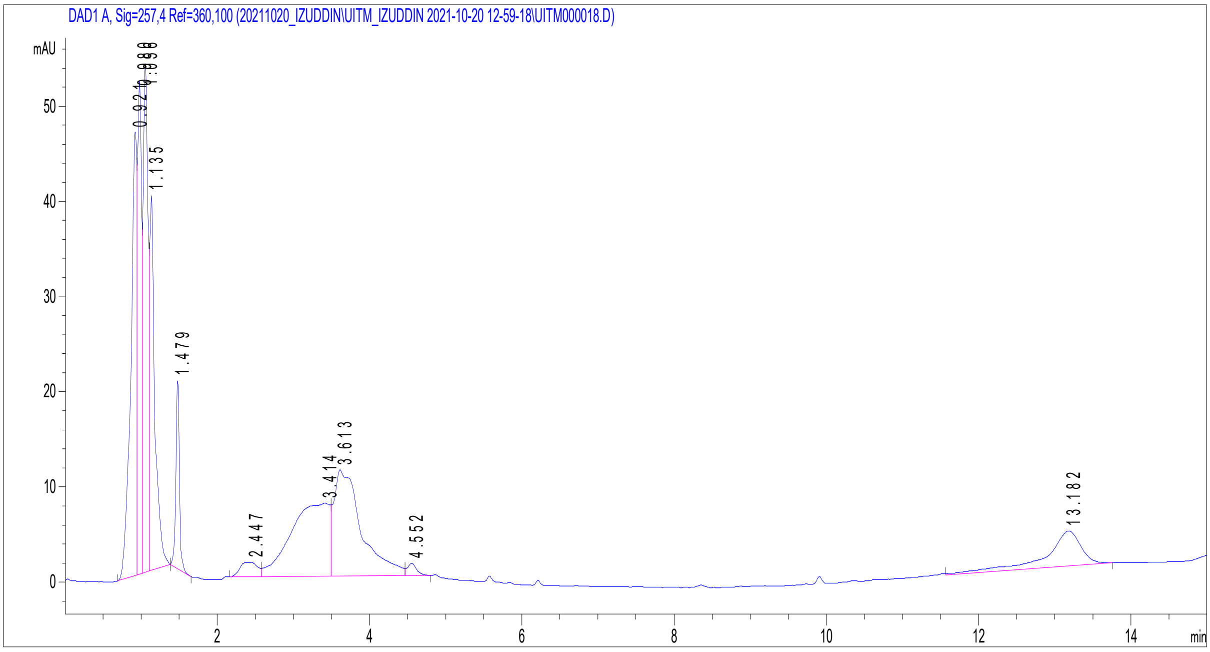

Figure S16. Chromatogram of BBHU60

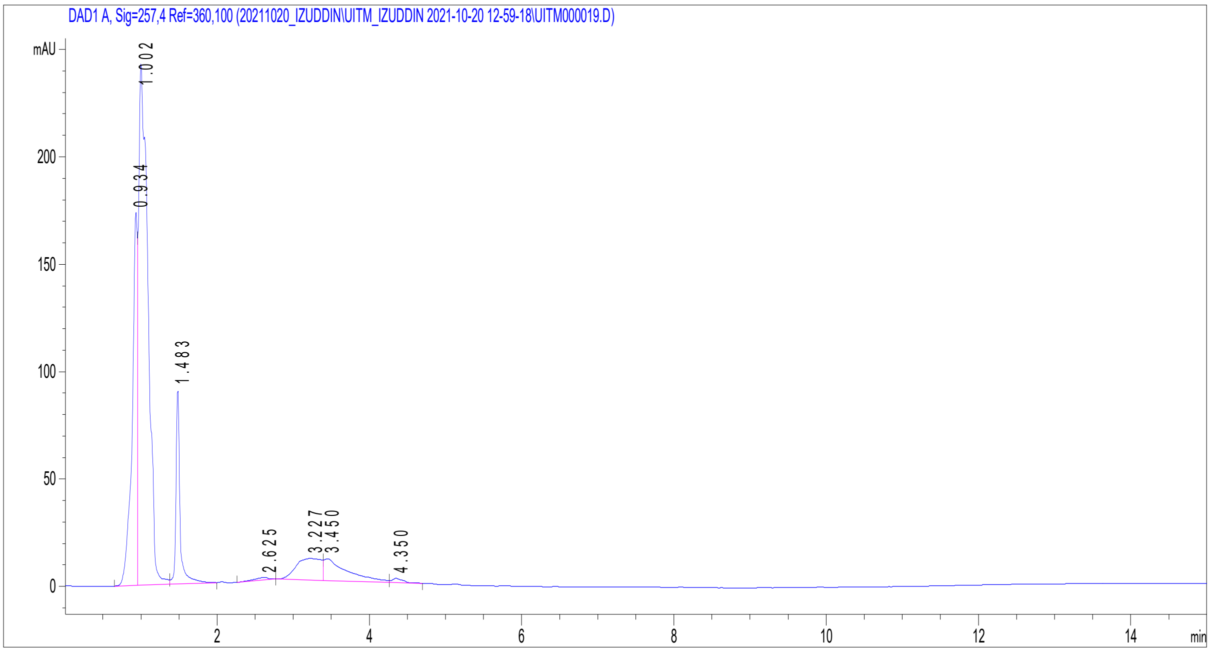


Figure S17. Correlation graph of TPC and DPPH free radical scavenging activity

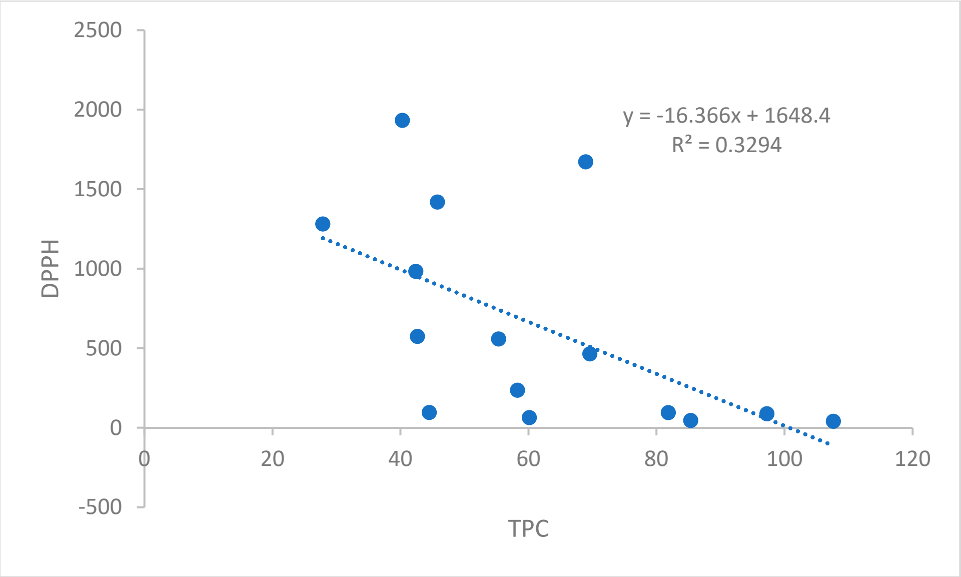

Figure S18. Correlation graph of TFC and DPPH free radical scavenging activity

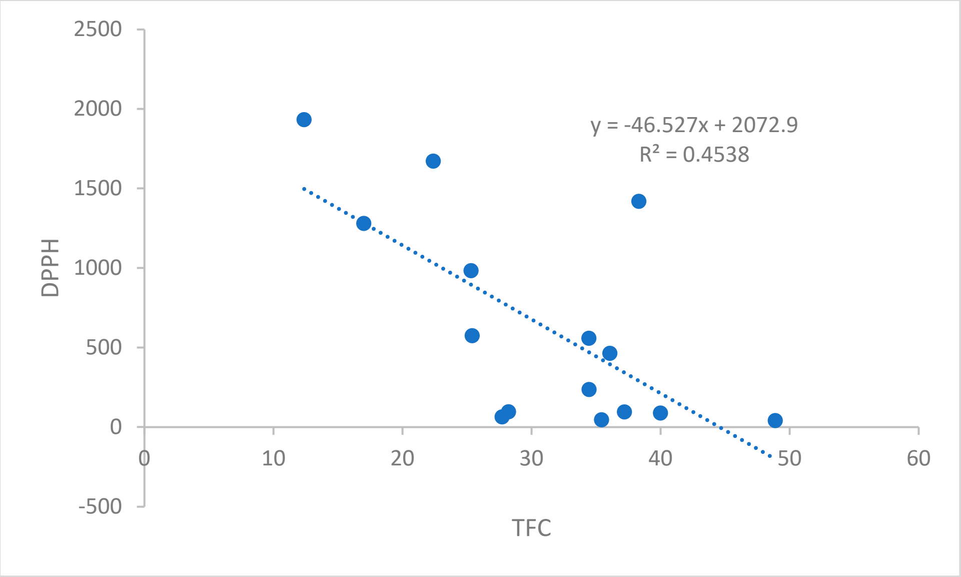

Supplement: Supplementary file 1 [file molecules-27-02359-s001.zip › molecules-1661623-supplementary.pdf]
